# Early recognition and mobilization of resources in managing amniotic fluid embolism for a high-risk obstetric patient: A case report

**DOI:** 10.1016/j.crwh.2024.e00634

**Published:** 2024-07-10

**Authors:** R. Zbeidy, Anh P. Le, Sarah M. Jacobs, Alexander W.M. Hall, P. Toledo

**Affiliations:** a University of Miami Miller School of Medicine, 1600 NW 10th Ave, Miami, FL 33136, United States of America; b Department of Anesthesiology, Jackson Memorial Hospital, 1611 NW 12^th^ Ave, Miami, FL 33136, United States of America

**Keywords:** Amniotic fluid embolism, Venoarterial extracorporeal membrane oxygenation, Point-of-care, Cesarean delivery, Obstetric

## Abstract

A 33-year-old woman, gravida 3 para 2, at 39 weeks of gestation, undergoing induction of labor, had a seizure. She was transferred to the operating room and underwent a cesarean delivery for non-reassuring fetal status. An amniotic fluid embolism (AFE) was suspected given her cardiovascular collapse, disseminated intravascular coagulation, and early right heart failure. Early mobilization of resources (e.g., blood bank, gynecology oncology, extracorporeal membrane oxygenation) was necessary as the hospital was in a stand-alone building. Biomarkers were sent during the acute event. The creation of an AFE order set is discussed.

## Highlights

• An amniotic fluid embolism (AFE) requires early recognition and the patient may need prompt cardiopulmonary resuscitation.
• Early mobilization of a team of surgeons, interventionalists, and specialty anesthesiologists is needed.
• Other critical actions include point-of-care echocardiography.
• An AFE order set should be created to aid diagnosis, define incidence, and identify risk factors.

## 1 Introduction

Amniotic fluid embolism (AFE) is a rare, but potentially life-threatening complication of pregnancy [1,2]. The clinical diagnosis is one of exclusion, as there are no universally accepted criteria to diagnose AFE [[3], [4], [5]]. The exact etiology of AFE also remains unclear, thus underscoring the importance of prompt initiation of high-quality supportive care for optimal maternal outcomes. AFE-related mortality has decreased in recent times, largely driven by advances in the management of critically ill parturients, but early mobilization of resources is critical when labor and delivery are distant from an operating room. This report describes the management of a case of suspected AFE with the goal of highlighting systems-level management elements for stand-alone labor and delivery units, and the creation of AFE order sets in order to further our understanding of this condition. This case report adheres to the applicable EQUATOR guidelines.

## 2 Case Presentation

A 33-year-old woman, gravida 3 para 2, at 39 of weeks gestation was admitted to the labor and delivery unit for induction of labor. Her past medical history was significant for well-controlled human immunodeficiency virus (HIV), with an undetectable viral load, uterine fibroids, and genital herpes simplex virus. Her home medications included bictegravir-emtricitabine-tenofovir, ferrous sulfate, ondansetron, and prenatal vitamins. Upon admission, there was a category I fetal heart tracing (FHT), normal vital signs, and laboratory findings were remarkable for only anemia (a hemoglobin level of 9.9 g/dl). Labor was induced with misoprostol and she later requested neuraxial labor analgesia. The obstetric team performed an artificial rupture of membranes following initiation of neuraxial labor analgesia, at which time the FHT changed to category 2. An intrauterine uterine pressure catheter (IUPC) was placed and amnioinfusion was started. Two hours after IUPC placement, the patient started seizing and a prolonged fetal deceleration was noted. Magnesium was initiated for presumed eclampsia and the patient was transferred to the operating room for a cesarean delivery.

Following pre‑oxygenation, a rapid sequence induction was performed. Four minutes after delivery of a live infant, pulseless electrical activity (PEA) occurred. Cardiopulmonary resuscitation was initiated with spontaneous return of spontaneous circulation (ROSC) after two minutes. Following the cardiac arrest, the patient had profound atony and postpartum hemorrhage. The massive-transfusion protocol was initiated. Over the next 55 min, the patient had five more episodes of PEA, each of which was followed by prompt ROSC. The gynecology oncology service, in addition to the cardiac anesthesiology team, and the extracorporeal membrane oxygenation (ECMO) team were activated.

**Fig. 1:**
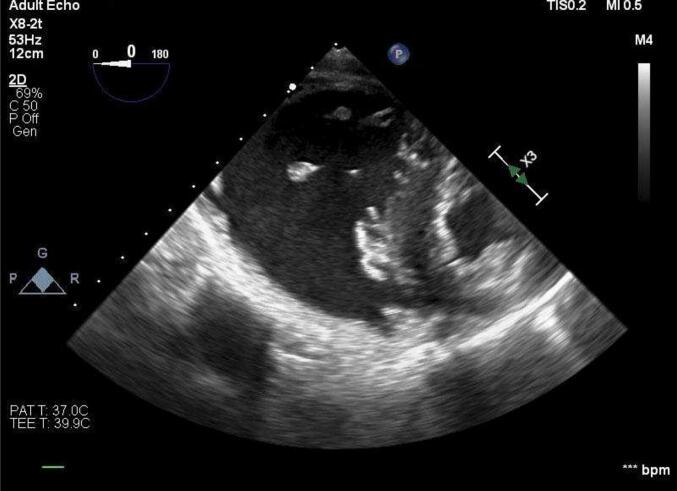
Transesophageal echocardiogram demonstrates dilated right ventricle indicating right heart strain.

Point-of-care transthoracic echocardiography demonstrated a severely dilated right ventricle and empty left ventricle. Given the clinical scenario, high suspicion was given to the diagnosis of AFE (Fig. 1). A profound coagulopathy ensued with a nadir fibrinogen of 119 mg/dL, an international normalized ratio increase of 33%, and a platelet count of 28,000 per microliter of blood. Due to the high suspicion for AFE, biomarkers for AFE, including tryptase, histamine, and complement, were sent to the laboratory. C3 and C4 levels were both depressed, with values of 40 mg/dL and 6 mg/dL, respectively, along with an elevated tryptase level of 13 μg/L.

Femoral VA-ECMO was initiated in the operating room 90 mins after the initial arrest. Postoperatively, prior to transfer to the ICU, the patient had an expanding abdomen and intrabdominal bleeding requiring an exploratory laparotomy and subsequent hysterectomy. The placement of a Bakri balloon for tamponade was not feasible due to the presence of ECMO cannula in the femoral artery and vein, a B-lynch suture was attempted, but it failed to stop the bleeding, necessitating the performance of a hysterectomy. The total estimated blood loss was 10 L She received two liters of crystalloid, 15 units of packed red blood cells, 15 units of fresh frozen plasma, 3 units of cryoprecipitate and 3 units of platelets.

On postoperative day (POD) 1 the patient continued to hemorrhage, requiring bilateral uterine artery embolization. Her intensive care unit course was complicated by acute kidney injury and transaminitis secondary to hypovolemic shock. On POD 3, she developed a saddle pulmonary embolism and underwent a thrombectomy. A pathology report of the uterus showed myometrial vessels with intraluminal amniotic fluid. She was successfully decannulated and extubated on POD 5 and made a rapid recovery without any permanent neurological defects. She was discharged on POD 14.

## 3 Discussion

Amniotic fluid embolism is a rare, catastrophic complication of pregnancy with estimated maternal mortality of 6–10% [6]. AFE is likely the least preventable of maternal mortalities, thus making timely initiation of resuscitative measures of critical importance. This case report demonstrates the importance of early recognition of AFE and the vital role of prompt advanced cardiovascular life support-guided CPR and mobilization of a multidisciplinary team of surgeons, interventionalists, and specialty anesthesiologists in a stand-alone women's hospital. Management of AFE requires immediate delivery, supportive therapy, correction of coagulopathy, and prompt cardiopulmonary resuscitation. The use of both transthoracic echocardiography and transesophageal echocardiography was useful, both to help exclude alternative diagnoses, such as pulmonary embolism and hypovolemia, and to guide clinical management intraoperatively. Additionally, as previous case reports have highlighted the importance of ECMO therapy, mobilization of the ECMO team was critical for cardiopulmonary support. The management of critical coagulopathy in the face of cardiogenic shock is challenging. Heparinization required to facilitate mechanical circulatory support during ongoing coagulopathy was managed using frequent POC TEG with heparinase to guide blood product resuscitation.

Amniotic fluid embolism has a classical presentation of 1) acute respiratory distress, 2) cardiovascular collapse, and 3) coagulopathy near the time of delivery [7]. Sudden loss of consciousness has been reported as the most common presentation in AFE, but cough, seizures, and other promontory signs have also been reported [8]. The patient's seizure was initially suspected to be associated with eclampsia, but the patient had no other signs or symptoms consistent with preeclampsia, and, given the timing of her cardiac arrest, which was followed by massive hemorrhage, the clinical diagnosis shifted to AFE.

The pathophysiology of AFE remains unknown, with most theories relating it to a systemic inflammatory response to fetal antigens causing an inappropriate release of endogenous inflammatory and vasoactive mediators [3]. To date, there is no definitive test to confirm the diagnosis of AFE. Histopathologic findings of embolic material of squamous cells, mucin, meconium, and amorphous eosinophilic material and laboratory findings of elevated tryptase, histamine, and depressed C3 and C4 have been indicated, but not specific to AFE [9,10]. Due to the suspicion for AFE, clinical biomarkers associated with AFE in the literature were drawn. C3 and C4 levels were both depressed, with values of 40 mg/dL and 6 mg/dL, respectively, along with an elevated tryptase level of 13 μg/L. While these did not result during the acute episode of care and are non-specific, they help support the clinical diagnosis. An AFE order set has since been created at this institution for providers who have a case of suspected AFE intraoperatively to assist diagnosis and help define the incidence and risk factors of a poorly characterized and devastating pathology.

In conclusion, we describe the successful management of a case of suspected amniotic fluid embolism in a stand-alone women's hospital. Systems-level actions critical to the recognition and management include the use of POC echocardiography to assist with diagnosis, activation of multidisciplinary teams, including an ECMO team, and the creation of an AFE order set. We hope that other institutions create a similar biomarker-based order set to further our understanding of this rare disease.

## Contributors

R. Zbeidy contributed to patient care, conception of the case report, drafting the manuscript and revising the article critically for important intellectual content.

Anh P. Le contributed to acquiring and interpreting the data, drafting the manuscript, undertaking the literature review and revising the article critically for important intellectual content.

Sarah M. Jacobs contributed to drafting the manuscript and undertaking the literature review.

Alexander W.M. Hall contributed to acquiring and interpreting the data and drafting the manuscript.

P. Toledo contributed to conception of the case report, drafting the manuscript and revising the article critically for important intellectual content.

All authors approved the final submitted manuscript.

## Funding

No funding from an external source supported the publication of this case report.

## Patient consent

Obtained.

## Provenance and peer review

This article was not commissioned and was peer reviewed.

## Conflict of interest statement

Paloma Toledo, M.D., M.P.H. is supported by a grant from the 10.13039/100000080Anesthesia Patient Safety Foundation and has received speaker fees from Paciri Biosciences, Int. The other authors declare that they have no conflict of interest regarding the publication of this case report.
